# Immunoglobulin G4–Related Disease of the Genitourinary System

**DOI:** 10.1097/og9.0000000000000171

**Published:** 2026-05-07

**Authors:** Meryl G. Warshafsky, Margaret A. Flanigan, Sarah G. Bell, Sharon A. Kreuer, Erika M. Moore, Shannon K. Rush

**Affiliations:** Division of Gynecologic Oncology, UPMC Magee-Women's Hospital, and the Department of Radiology, UPMC, Pittsburgh, Pennsylvania; the Division of Gynecologic Oncology, University of Michigan, Ann Arbor, Michigan; and the Department of Pathology, Cleveland Clinic Foundation, Cleveland, Ohio.

## Abstract

**BACKGROUND:**

Immunoglobulin G4–related disease (IgG4-RD) is an immune-mediated disease that is often misdiagnosed given its rare incidence and misleading presentation. Prior case studies have described IgG4-RD involving the ovaries, uterus, and bladder. This article describes the first case of vaginal IgG4-RD.

**CASE:**

A 76-year-old woman presented with episodes of vaginal spotting, vaginal cuff friability, and benign inflammation on vaginal pathology. She was unsuccessfully treated with topical estrogen and antibiotics. She ultimately was found to have a progressive, obstructive pelvic mass near the vaginal cuff that was surgically excised, with mass pathology showing lymphoplasmacytic disease. The mass recurred and repeat sampling of the vaginal apex confirmed IgG4-RD.

**CONCLUSION:**

This report showcases the novel finding of vaginal IgG4-RD. The patient's decade-long course before diagnosis highlights the challenges in identifying this disease. This case report aims to increase awareness of vaginal IgG4-RD, leading to timely diagnosis and treatment.

## Abstract 2

Vaginal immunoglobulin G4–related disease can mimic a gynecologic neoplasm and should be considered in the differential diagnosis of recurrent vulvovaginal masses.

**Teaching Points**

1. Steroids are the primary treatment in patients without known contraindications.
2. For persistent, benign vulvovaginal masses, consider work-up for genitourinary immunoglobulin G4–related disease.

Immunoglobulin G4–related disease (IgG4-RD) is a rare, immune-mediated disease that involves one or more organs and manifests in many ways (Appendix 1, available online at http://links.lww.com/AOG/E647). Immunoglobulin G4-RD has an estimated incidence of 0.78–1.39 per 100,000 person-years^1^ and often presents with a tumor in an affected organ or with diffuse enlargement of an organ. This disease is pathologically recognized by the presence of a dense lymphoplasmacytic infiltrate with IgG4-positive plasma cells, storiform fibrosis, and tissue eosinophilia. Serum IgG4 is elevated in two-thirds of patients but is not diagnostic of IgG4-RD. Immunoglobulin G4-RD is frequently misdiagnosed given that its presentation mimics neoplasm or infection. Diagnosis is confirmed when a patient meets two criteria: 1) clinical or radiologic evidence of a tumor in an involved organ and, 2) biopsy of that organ demonstrates histologic findings associated with IgG4-RD. Response to treatment with systemic glucocorticosteroids is dramatic and is often suggested as a diagnostic criterion.

## CASE

A 76-year-old woman with a medical history of hypertension, hypothyroidism, and hyperlipidemia presented in 2013 with vaginal bleeding. She had undergone benign vaginal hysterectomy in 1976 and had no problems until 2010, when she presented to her gynecologist with scant vaginal spotting. At that time on examination, she had a small area of friability at the vaginal apex that was cauterized with silver nitrate. She had no additional bleeding until May 2013, when she presented for evaluation of new, bright red vaginal bleeding. She had repeated examinations that demonstrated apical friability. At the time of these examinations, she underwent multiple nondiagnostic Pap tests, at which time she was referred to gynecologic oncology for further work-up. Her initial examination after referral was notable for possible fine nodularity at the vaginal apex. Given this nodularity, she underwent an examination under anesthesia with vaginal biopsies in 2013 that showed severe acute and chronic inflammation with granulation tissue. She was subsequently treated with vaginal estrogen.

Months after topical estrogen use, a repeat examination was notable for worsened circumferential nodularity from the vaginal apex to the mid-third of the vagina. Biopsies performed both in the office and in the operating room again showed severe chronic inflammation and granulation tissue. Given the persistence of inflammation without evidence of malignancy, she was referred to the reproductive infectious disease department, where she was treated with a prolonged course of topical clindamycin for a presumptive diagnosis of desquamative inflammatory change. Her symptoms were stable but not improved after this course of antibiotics.

**Fig. 1.**
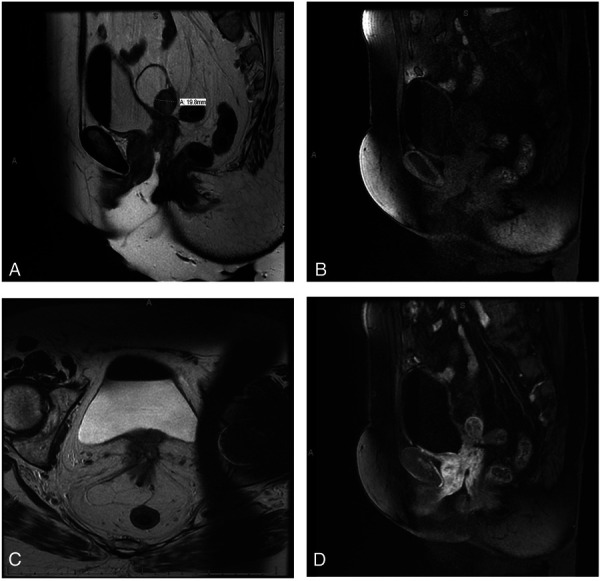
Magnetic resonance imaging of the pelvis showing involvement of ovary and vaginal cuff (**A–C**) and degree of postcontrast enhancement (**D**).

She next returned for evaluation approximately 18 months later. At that time, pelvic ultrasonography demonstrated a complex cystic structure in the midline of the pelvis at the vaginal cuff, measuring 4×4.2×3.7 cm. On retrospective review of previous computed tomography imaging obtained in April 2013, it appeared that this structure had been present previously, and the extent of interval growth was unclear. Pelvic magnetic resonance imaging (MRI) was therefore obtained and revealed a complex solid and cystic mass at the vaginal apex involving both ovaries, as well as diffuse nodular thickening and enhancement of the vagina and urethra (Fig. 1). Repeated biopsies continued to demonstrate severe acute and chronic inflammation and granulation tissue with insufficient quantity of IgG4-positive plasma cells to be diagnostic of IgG4-RD. Given that there was no evidence of malignancy, the patient was counseled on options for expectant management with serial imaging or surgical management.

*Warshafsky. Genitourinary IgG4-RD. O&G Open 2026*.

She opted for expectant management until 2016, when she developed new urinary retention and bilateral hydronephrosis. Repeat pelvic MRI at that time suggested that her retention and hydronephrosis were secondary to the inflammatory process in her pelvis. Therefore, the patient opted to undergo radical cystectomy, vaginectomy, bilateral oophorectomy, and creation of an ileal conduit. Final pathology from her surgery showed xanthogranulomatous lymphoplasmacytic inflammation without comment on IgG4 reactivity.

**Fig. 2.**
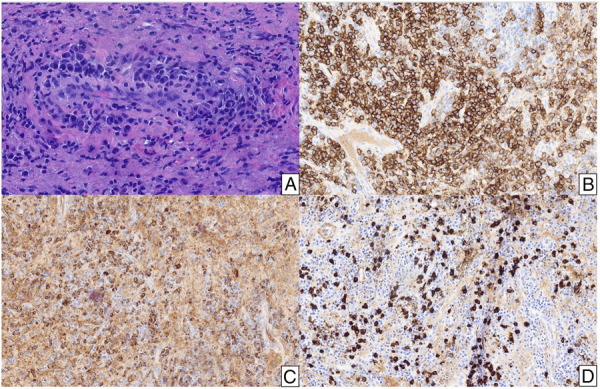
A hematoxylin and eosin section shows increased plasma cells (×400 magnification) (**A**). A CD138 stain confirms the presence of many plasma cells (×200 magnification) (**B**), and additional stains for immunoglobulin G (IgG) (×200 magnification) (**C**) and IgG4 (×200 magnification) (**D**) demonstrate increased IgG4-positive plasma cells, which comprise more than 40% of the IgG-positive plasma cells.

The patient remained without issue until 2023, when she presented to her gynecologist with daily vaginal bleeding. She returned to gynecologic oncology and was noted to have a 4-cm erythematous, polypoid, friable mass that completely replaced her vaginal apex. This mass was closely associated with the rectum. Additional biopsies of the vaginal apex performed at that time revealed fibrous tissue with prominent lymphoplasmacytic infiltrate and increased IgG4-positive plasma cells without evidence of malignancy (Fig. 2). Based on these biopsies, the hematopathologist felt this was consistent with IgG4-RD. Prior specimens were not retrospectively reviewed.

*Warshafsky. Genitourinary IgG4-RD. O&G Open 2026*.

Given the concern for IgG4-RD, the patient was referred to a rheumatologist, who conducted a laboratory assessment that included a serum IgG4 level, complement 3 and 4 testing, an antineutrophil cytoplasmic antibodies screen, and C-reactive protein, which were all normal. However, given her pathology results, she was started on prednisone 40 mg daily with a plan to taper steroids over the course of 3 months. She noted a cessation in her vaginal bleeding within 3 days of starting steroids. On repeat pelvic examination 4 months after starting the steroids, she had almost complete resolution of the friable lesion at her vaginal apex. She was told that a disease-modifying antirheumatic drug would be used if she had a recurrence of vaginal bleeding. Since then, she has followed up with a rheumatologist for serologic monitoring every 3 months and with a gynecologic oncologist for pelvic examinations every 6 months. In December 2025, she had new vaginal bleeding and inflammatory changes on pelvic examination. She repeated a prednisone taper with resolution of symptoms (Appendix 2, available online at http://links.lww.com/AOG/E647).

## DISCUSSION

To our knowledge, this is the first report of direct vaginal involvement in IgG4-RD; previous isolated case studies have described IgG4-RD that involved the ovaries, uterus, and bladder.^2–11^ Although renal involvement in IgG4-RD is not unusual, IgG4-RD of the lower urinary and genital organs is significantly less common.^12,13^

Due to its relative rarity, IgG-RD can be difficult to diagnose—as demonstrated in this case. Classification of both inclusion and exclusion criteria for IgG4-RD were recently agreed upon by the American College of Rheumatology and the European League Against Rheumatism, which resulted in clearly defined clinical, serologic, pathologic, and radiologic characteristics of this disease.^14^ The inclusion criteria are positive immunostaining and clinical or radiologic involvement of a common anatomy, such as the head and neck, chest, pancreas and biliary tree, kidney, and retroperitoneum. Exclusion criteria are defined along five categories, which include clinical, serologic, radiologic, pathologic, and specific disease exclusions.

When applying these criteria to this case, the patient had ureterovesical involvement as demonstrated on pelvic MRI. Although IgG4-RD is known to involve the kidney, this case demonstrates urologic disease secondary to direct extension from the pelvis. Primary pelvic disease is not listed under the inclusion criteria, making vaginal origin a novel finding. The exclusion criteria for IgG4-RD include the absence of malignancy. This patient had multiple pathologic specimens that demonstrated inflammation without evidence of malignancy. However, undiagnosed IgG4-RD can lead to organ dysfunction and subsequent failure, likely what led to our patient's initial cystectomy and vaginectomy. Additionally, the lack of an objective response to glucocorticoid therapy is a reason to suspect a cause unrelated to IgG4-RD. In this patient, treatment with daily prednisone resulted in near complete resolution of her vaginal mass. Refractory vulvovaginal disorders can be successfully treated with oral corticosteroids and symptom resolution may not prompt further diagnosis.

Steroids are the primary treatment for IgG4-RD in patients without known contraindications. Response to treatment often occurs acutely and within 2 to 4 weeks of starting steroid use. Patients can experience improvement in symptom burden, drastic reduction in size of discrete tumor or involved organ, and rapid improvement in organ dysfunction. In our case, the patient experienced symptom improvement within 1 week and had repeat imaging 1 month after completing the steroid course, which showed near resolution of the tumor. However, long-term steroid use is insufficient to control persistent or recurrent disease and has its own risks associated with prolonged use. Relapse of IgG4-RD is common, occurring in 24–54% of patients.^15^ Many studies have investigated clinical and pathologic predictors of recurrence including serum IgG4 and IgE levels as well as multi-organ involvement. Rituximab is indicated for recurrent disease but may also be considered as a component of primary treatment in patients with characteristics suggestive of unremitting disease. Combination primary therapy is debated among experts, but it may benefit certain patient populations. Rituximab and other disease-modifying antirheumatic drugs are the mainstay of secondary treatment and would be indicated for our patient should she develop recurrence.

IgG4-RD is an immune-mediated disease that can involve any organ and lead to fibro-inflammatory changes and organ dysfunction. This case report serves to describe a unique case of IgG4-RD that originated in the pelvis with proximal progression. Due to the unique presentation, accurate diagnosis and subsequent treatment initiation were delayed. Inclusion and exclusion criteria have been identified with notable emphasis on response to steroid treatment. This case highlights the importance of adding genitourinary IgG4-RD work-up for persistent, yet benign, vulvovaginal masses.

## References

[1] Wallace ZS Miles G Smolkina E Petruski-Ivleva N Madziva D Cook C Incidence, prevalence and mortality of IgG4-related disease in the USA: a claims-based analysis of commercially insured adults. Ann Rheum Dis 2023;82:957–62. doi: 10.1136/ard-2023-223950 37137671

[2] Senda Y Ikeda Y Tamauchi S Yoshikawa N Kikkawa F Kajiyama H. A uterine pseudotumor of immunoglobulin G4-related disease. J Obstet Gynaecol Res 2021;47:430–5. doi: 10.1111/jog.14531 33059388

[3] Pacyna RR Cipriani NA Mathew MS Kim JS. IgG4-related disease mimicking gynecologic malignancy. Gynecol Oncol Rep 2023;45:101137. doi: 10.1016/j.gore.2023.101137 36714372 PMC9879761

[4] Alorjani MS Obeidat NA Ababneh EI Salem AA Matalka II. A 47-year-old woman with immunoglobulin G4 (IgG4)-related disease involving the right ovary. Am J Case Rep 2020;21:e926803. doi: 10.12659/AJCR.926803 33108358 PMC7603799

[5] Maruyama S Sato Y Taga A Emoto I Shirase T Haga H. Immunoglobulin G4-related disease presenting as bilateral ovarian masses and mimicking advanced ovarian cancer. J Obstet Gynaecol Res 2016;42:103–8. doi: 10.1111/jog.12835 26461453

[6] Akyol S Atalay F Hasdemir S Yerci Ö. IgG4-Related disease of the ovary. Turk Patoloji Derg 2021;37:63–6. doi: 10.5146/tjpath.2020.01500 32779156 PMC10508929

[7] Oh JW Rha SE Choi MH Oh SN Youn SY Choi JI. Immunoglobulin G4-related disease of the genitourinary system: spectrum of imaging findings and clinical-pathologic features. Radiographics 2020;40:1265–83. doi: 10.1148/rg.2020200043 32870766

[8] Cicioni G Iannone I Crocetti D Padua C Coppola A Petramala L . IgG4-related disease of the retroperitoneum mimics malignancy: a case report and literature review. In Vivo (Athens, Greece) 2025;39:2154–64. doi: 10.21873/invivo.14011 40579033 PMC12223607

[9] Fang Z Sun Y Ming S Li L Gao X. IgG4-related disease of the ureter mimicking malignant ureter tumor: a case report and experience sharing. AME Case Rep 2024;8:4. doi: 10.21037/acr-23-33 38234351 PMC10789895

[10] Nguyen T Brodsky S Maroz N. Progression to end-stage renal disease due to IgG4-Related nephritis refractory to rituximab. Cureus 2023;15:e36327. doi: 10.7759/cureus.36327 37077588 PMC10108657

[11] Vijayvergia P Mukherjee S Singh L Dhakad U. Urinary bladder involvement in IgG4-related disease: a case-based review. Mod Rheumatol Case Rep 2024;8:344–7. doi: 10.1093/mrcr/rxae011 38537149

[12] Mbengue M Goumri N Niang A. IgG4-related kidney disease: pathogenesis, diagnosis, and treatment. Clin Nephrol 2021;95:292–302. doi: 10.5414/CN110492 33860756

[13] Boffa JJ Esteve E Buob D. Renal involvement in IgG4-related disease. Presse Med 2020;49:104017. doi: 10.1016/j.lpm.2020.104017 32234380

[14] Yoo BW Song JJ Park YB Lee SW. 2019 American College of Rheumatology/European League Against Rheumatism classification criteria for IgG4-related disease by Wallace. Ann Rheum Dis 2022;81:e179. doi: 10.1136/annrheumdis-2020-217086 32054605

[15] Zongfei J Lingli C Ying S Lingying M Lijuan Z Dongmei L Clinical and pathological predictors of relapse in IgG4-related disease. Arthritis Res Ther 2022;24:106. doi: 10.1186/s13075-022-02792-z 35546243 PMC9092827

